# Carotenoids and amphibians: effects on life history and susceptibility to the infectious pathogen, *Batrachochytrium dendrobatidis*

**DOI:** 10.1093/conphys/cov005

**Published:** 2015-03-13

**Authors:** Rickey D Cothran, Stephanie S Gervasi, Cindy Murray, Beverly J French, Paul W Bradley, Jenny Urbina, Andrew R Blaustein, Rick A Relyea

**Affiliations:** 1 Department of Biological Sciences, Southwestern Oklahoma State University, Weatherford, OK 73096, USA; 2 Department of Integrative Biology, University of South Florida, Tampa, FL 33620, USA; 3 Department of Biological Sciences, University of Pittsburgh, Pittsburgh, PA 15260, USA; 4 Environmental Sciences Graduate Program, Oregon State University, Corvallis, OR 97331, USA; 5 Department of Biological Sciences, Rensselaer Polytechnic Institute, Troy, NY 12180, USA

**Keywords:** Amphibian decline, disease ecology, nutritional ecology, parasite, pathogen

## Abstract

Carotenoids are considered beneficial nutrients because they provide increased immune capacity. Although carotenoid research has been conducted in many vertebrates, little research has been done in amphibians, a group that is experiencing global population declines from numerous causes, including disease. We raised two amphibian species through metamorphosis on three carotenoid diets to quantify the effects on life-history traits and post-metamorphic susceptibility to a fungal pathogen (*Batrachochytrium dendrobatidis*; *Bd*). Increased carotenoids had no effect on survival to metamorphosis in gray treefrogs (*Hyla versicolor*) but caused lower survival to metamorphosis in wood frogs [*Lithobates sylvaticus* (*Rana sylvatica*)]. Increased carotenoids caused both species to experience slower development and growth. When exposed to *Bd* after metamorphosis, wood frogs experienced high mortality, and the carotenoid diets had no mitigating effects. Gray treefrogs were less susceptible to *Bd*, which prevented an assessment of whether carotenoids could mitigate the effects of *Bd*. Moreover, carotenoids had no effect on pathogen load. As one of only a few studies examining the effects of carotenoids on amphibians and the first to examine potential interactions with *Bd*, our results suggest that carotenoids do not always serve amphibians in the many positive ways that have become the paradigm in other vertebrates.

## Teaser

Carotenoids are components of an animal’s diet that are considered beneficial because they typically provide increased immune capacity. However, little research has been done in amphibians. We found that carotenoids can cause lower survival, slower development and growth in amphibians, and no mitigating effects against a common amphibian disease.

## Introduction

Animal diets provide energy and nutrients that are essential to life. Carotenoids are one such group of nutrients; they consist of more than 600 pigments that are categorized as either carotenes or xanthophylls (both are comprised of hydrocarbon chains, but carotenes contain no oxygen whereas xanthophylls contain oxygen; Pérez-Rodríguez, 2009). Carotenoids function as accessory pigments for photosynthesis and they are synthesized by plants and some species of bacteria and fungi; however, animals cannot synthesize carotenoids and must acquire them from their diet. In addition to producing colours in many animals (commonly in shades of yellow, orange or red), carotenoids are important antioxidants and immune system enhancers (Chew, 1993; Hill, 1995; Vershinin, 1999; Alonso-Alvarez *et al.*, 2004; but see Costantini *et al.*, 2007; Pérez-Rodríguez, 2009). As a result, animals fed diets higher in carotenoids should be less susceptible to many pathogens and diseases; this prediction has been supported in a number of animal species, including humans (Bendich, 1993; Skarstein and Folstad, 1996; Horak *et al.*, 2001; Hill and Farmer, 2005; Babin *et al.*, 2010).

There is broad agreement regarding the benefits of carotenoids, but there is debate over whether carotenoids can cause fitness costs (reviewed by Olson and Owens, 1998). If carotenoids are rare, it may be costly to find and consume a high-carotenoid diet. If carotenoids are abundant, animals may face a cost of consuming a diet that is too high in carotenoids. Excess carotenoids might also be costly if they are directly toxic, if they require a substantial amount of energy to detoxify them or if the breakdown products are toxic (Olson and Owens, 1998; Hartley and Kennedy, 2004). Studies confirming toxicity are rare (Kalariya *et al.*, 2009), but detrimental effects would be more likely to be observed in herbivores than in carnivores because the diets of herbivores typically contain more carotenoids per unit mass than the diets of carnivores (Olson and Owens, 1998).

Compared with other vertebrates, little is known about the effect of carotenoids on amphibians. Researchers have examined how carotenoids contribute to amphibian colouration (Forbes *et al.*, 1973; Frost and Robinson, 1984; Matsui *et al.*, 2003; Bell and Zamudio, 2012) and how such colours may serve to attract mates (Gomez *et al.*, 2009; Richardson *et al.*, 2009); however, only three studies have manipulated carotenoid in the diet. Ogilvy *et al*. (2012) demonstrated that diets containing different carotenoid concentrations affected red-eyed treefrogs (*Agalychnis callidryas*); increased carotenoids did not affect larval growth or survival, but they did cause greater post-metamorphic growth, redder skin and increased fecundity. In a second study, Ogilvy and Preziosi (2012) manipulated carotenoid diets in western clawed frogs [*Silurana* (*Xenopus*) *tropicalis*]; increased carotenoids caused more rapid larval development. Finally, in a third study, Dugas *et al*. (2013) discovered that increased carotenoids in diets resulted in higher reproductive success of captive-bred strawberry poison frogs (*Oophaga pumilio*).

Despite the important role that carotenoids play in animal immunity, no amphibian studies have examined the effect of carotenoids on amphibian diseases, yet this is an important question given the diversity of pathogens that harm amphibians (Blaustein *et al.*, 2011). One of the most lethal diseases in amphibians is chytridiomycosis, which is caused by the chytrid fungus *Batrachochytrium dendrobatidis* (hereafter termed ‘*Bd*’). The fungus grows on tissues containing keratin, which include the mouthparts of tadpoles and the skin of post-metamorphic frogs (Marantelli *et al.*, 2004; Berger *et al.*, 2005). The fungus is found throughout the world and is associated with declines in numerous species (Stuart *et al.*, 2004; Skerratt *et al.*, 2007; Fisher *et al.*, 2009; Olson *et al.*, 2013). Studies suggest that both innate and acquired immunity play a role in determining variation in host responses to *Bd*; for example, the innate immune system releases antimicrobial peptides and lysozymes onto the skin that can prevent or reduce the amount of *Bd* infecting amphibian skin cells (Rollins-Smith, 2009). In addition, bacteria growing on amphibian skin can produce antifungal chemicals that may kill *Bd* (Brucker *et al.*, 2008; Antwis *et al.*, 2014).

When *Bd* infects the epidermis, the fungus may inhibit lymphocyte proliferation (Rollins-Smith *et al.*, 2011; Fites *et al.*, 2013). While several studies have demonstrated that certain aspects of the acquired immune system defend against *Bd* infections (Richmond *et al.*, 2009; Ramsey *et al.*, 2010; Savage and Zamudio, 2011), others have not observed an adaptive immune response (Ribas *et al.*, 2009; Cashins *et al.*, 2013). There is general agreement that carotenoids can enhance both the acquired immune system, by increasing lymphocyte development, and the innate immune system, by improving the performace of antigen-presenting cells and serving as an important component for the epithelial cells that produce antimicrobial peptides (see reviews by Chew and Park, 2004; Duriancik *et al.*, 2010; Hall *et al.*, 2011). Given the common role that carotenoids play in stimulating the immune systems of vertebrates, it is important to know whether enhanced carotenoid diets in amphibians might make them more resistant or tolerant to *Bd* infections. As a result, we tested whether increasing the amount of carotenoids in the diet of amphibians improves tadpole growth and development and decreases the susceptibility of metamorphs to *Bd*.

## Materials and methods

### Experimental design

We performed identical experiments with two anuran species, namely wood frogs [*Lithobates sylvaticus* (*Rana sylvatica*)] and gray treefrogs (*Hyla versicolor*). For each species, we used a completely randomized design with a factorial combination of three carotenoid diets (none, low or high) fed during the larval and juvenile stages crossed with a post-metamorphic *Bd* exposure treatment (exposed or not exposed). The larval experiment was conducted at the Donald S. Wood Field Laboratory at the University of Pittsburgh Pymatuning Laboratory of Ecology; the post-metamorphic exposure to *Bd* was conducted at Oregon State University (OSU).

### Anuran collection and husbandry

In spring 2012, we collected the anurans as recently laid egg masses (seven masses of wood frogs, collected on 14 March) or amplecting adult pairs that subsequently laid eggs (15 masses of gray treefrogs, collected on 9 May). Eggs were kept in 90 l wading pools filled with well water until they hatched. After hatching, animals were fed rabbit chow (Blue Seal Bunny 16) *ad libitum* until used in experiments (approximately 1–2 weeks). The number of egg clutches, initial tadpole mass and initial developmental stages are presented in the Supplementary material appendix (Table A1).

### Experimental set-up

In each experiment, groups of eight tadpoles (drawn from a mixture of all egg masses) were randomly assigned to 14 l plastic tubs and reared on the three carotenoid diets in laboratory conditions [15 h–9 h light–dark photoperiod at a temperature of 22 ± 0.02 (mean ± 1 SD)°C]. As detailed in in the Supplementary material appendix (Table A1), the tadpoles began as recent hatchlings that were small in mass and early in development. Each tub contained 10 l of carbon-filtered, ultraviolet-irradiated water. The three carotenoid treatments were replicated 10 times for wood frogs and 12 times for gray treefrogs. To prevent water fouling, we changed the water in the tubs every 3–4 days when the tadpoles were young and small and every 2 days as they grew into large tadpoles. We raised all tadpoles on a base diet containing fishmeal, wheat flour, rice flour and vitamins (for similar diets fed to amphipods and tadpoles, see Babin *et al*., 2010; Ogilvy and Preziosi, 2012). This plant- and animal-based diet is appropriate because tadpoles can be omnivorous (Schiesari *et al.*, 2009).

We manipulated the carotenoid content of the diets (1 or 10 mg of carotenoids/g of total dry ingredients) by varying the amount of astaxanthin and lutein (details in the Supplementary material appendix; Tables A2 and A3). To our knowledge, there is no literature on the specific carotenoids found in foods (e.g. periphyton) consumed by wood frog and gray treefrog tadpoles; however, astaxanthin and lutein are commonly found in large quantities in both invertebrate and vertebrate aquatic animals, with the former generally being more prevalent than the latter (Matsuno, 2001; Guerin *et al.*, 2003; Gaillard *et al.*, 2004). On the basis of a previous study on amphibians (Ogilvly and Preziosi, 2012), we chose 10 mg of carotenoid per 1 g of food as our high-carotenoid diet. Such a diet is not unreasonable given that algae, the typical source of carotenoids in aquatic systems, can have carotenoid concentrations >30 mg/g dry biomass (Olaizola and Huntley, 2003).

The carotenoid manipulations were maintained throughout the larval period and after metamorphosis. The tadpoles were fed fresh food *ad libitum* until they developed forelimbs, at which point they were transferred to 14 l plastic tubs lined with damp sphagnum moss. Individuals were considered to have completed metamorphosis once they had fully absorbed their tail. We defined the time to metamorphosis as the time elapsed from the start of the experiment until full tail resorption. Once the tadpoles metamorphosed, we fed the metamorphs an *ad libitum* diet of crickets (*Acheta domesticus*) that had been reared on the same carotenoid diets as the tadpoles. Details regarding cricket maintenance can be found in the Supplementary material appendix.

Once all animals metamorphosed, we shipped them to OSU for *Bd* exposure. For reasons of shipping, we pooled animals from the same carotenoid treatment and thus lost tub-level identity when analysing for an effect of carotenoid diet on mass. As a result of asynchronous metamorphosis both within and among carotenoid treatments, the metamorphs were held for a maximum of 33 and 43 days for wood frogs and gray treefrogs, respectively, before being shipped to OSU.

After arrival at OSU, all metamorphs were transferred to glass terraria and maintained between 20 and 22°C on a 13 h–11 h light–dark photoperiod. The animals were acclimated to these conditions for 24 h before we initiated experiments. All animals were weighed and then randomly assigned to individual Petri dishes (140 mm × 30 mm), which contained 15 ml of dechlorinated water and had holes in the lid. When quantifying individual mass, we did not weigh each individual on the day it metamorphosed, but instead weighed all animals after they were transported to OSU and immediately before applying the *Bd* treatment. Once they arrived, the metamorphs continued to be fed crickets that were reared on the different carotenoid diets.

An equal number of animals from each carotenoid treatment were randomly assigned to the *Bd* and no *Bd* exposure treatments. Water in the Petri dishes was changed on day 7, and the Petri dishes were not reinoculated. We used the *Bd* isolate JEL 274; additional details regarding *Bd* preparation and inoculation can be found in the Supplementary material appendix.

During the *Bd* exposure, all animals were fed pinhead crickets twice per week under a ration that was related to metamorph mass (one cricket for every 0.1 g of average body weight by species). These crickets were reared using the same range of carotenoid diets as described above.

We monitored animals daily for signs of mortality. In accordance with our Animal Care and Use protocol, we quantified dead animals as those that either died or showed signs of distress (i.e. loss of righting reflex, failure to respond to stimulus, and seizures); any moribund animals were euthanized. Animals that died were immediately removed and individually stored in 95% ethanol to preserve *Bd* DNA for later analysis. At the end of the experiment, all remaining animals were killed in MS-222 and individually preserved in 95% ethanol.

### Assessing *B. dendrobatidis* infection load

To assess the *Bd* infection load, we swabbed the ventral abdominal skin and inner thigh skin using fine-tipped sterile rayon swabs (Medical Wire and Equipment, MW&E 113). For each species, we randomly sampled eight *Bd*-exposed animals from each carotenoid treatment (i.e. 24 animals in total). We also randomly sampled three control animals of each species to ensure that there was no contamination across treatments. Given that many of the wood frogs died but few of the gray treefrogs died, we sampled the *Bd* loads from the pool of dead and surviving frogs at the end of the experiment. All control animals tested negative for infection.

We used quantitative-PCR (qPCR) to assess the infection load in post-metamorphic amphibians following the methods of Boyle *et al.* (2004), except that we used 60 μl of Prepman Ultra (Applied Biosystems) instead of 40 μl in all DNA extractions. Extractions were diluted 1:10 and processed in an ABI PRISM 7500 (Applied Biosystems). Each sample was analysed in triplicate, and the mean number of genome equivalents of *Bd* per animal was calculated. Animals testing negative in at least two of the three replicate wells were considered ‘infection negative’.

### Statistical analyses

We conducted separate analyses for the wood frogs and gray treefrogs because the experiments for each species were separated in time (SPSS v. 21 for Mac). To test the effect of carotenoid treatment on survival to metamorphosis, we used a generalized linear model (GENLIN) that included a binomial probability distribution with a logit link function. To test for an effect of carotenoid diet on the time to metamorphosis, we used a GENLIN that included a Poisson probability distribution and a log-link function. In both models, the tub in which the tadpoles were raised was included as a subject variable to account for correlated responses among individuals within tubs. If we found a significant treatment effect in either model, we made pairwise comparisons using sequential Bonferroni tests. To test whether carotenoid diet affected the final mass of the animals immediately before the *Bd* exposure stage of the experiment, we used a generalized linear model. We used the MIXED procedure in SPSS, even though we do not have a random effect, because it employs a maximum likelihood estimation procedure that produces better parameter estimates for unbalanced designs.

To examine how metamorph survival was affected by *Bd* exposure, carotenoid diet and their interaction, we used a Cox regression. Mass was included as a covariate in the analysis because mass often correlates with susceptibility to *Bd* (Searle *et al*., 2011). Given that none of the second- or third-order interactions with mass was significant (all *P* ≥ 0.2), we dropped these terms from the analysis. We also ran the model without mass included, and the interpretation of the results was qualitatively the same.

To test for differences in infection load, we conducted an analysis of variance (ANOVA) on the mean number of genome equivalents of *Bd* per animal. Our initial analyses included mass as a covariate, but mass and the carotenoid-by-mass interactions were not significant, so we excluded these terms from the final analysis. For the gray treefrogs, we removed two data points that were extreme statistical outliers (one from a low-carotenoid treatment and the other from a high-carotenoid treatment). One individual was removed because it had a very high standard deviation among the three swab samples for *Bd* load (mean = 14.7, SD = 19.38). The other individual was removed after inspecting the box plots; it was three times the interquartile range. Excluding these two data points did not change the interpretation of our results.

## Results

### Effects of carotenoid diets on life-history traits before *B. dendrobatidis* exposure

**Figure 1:**
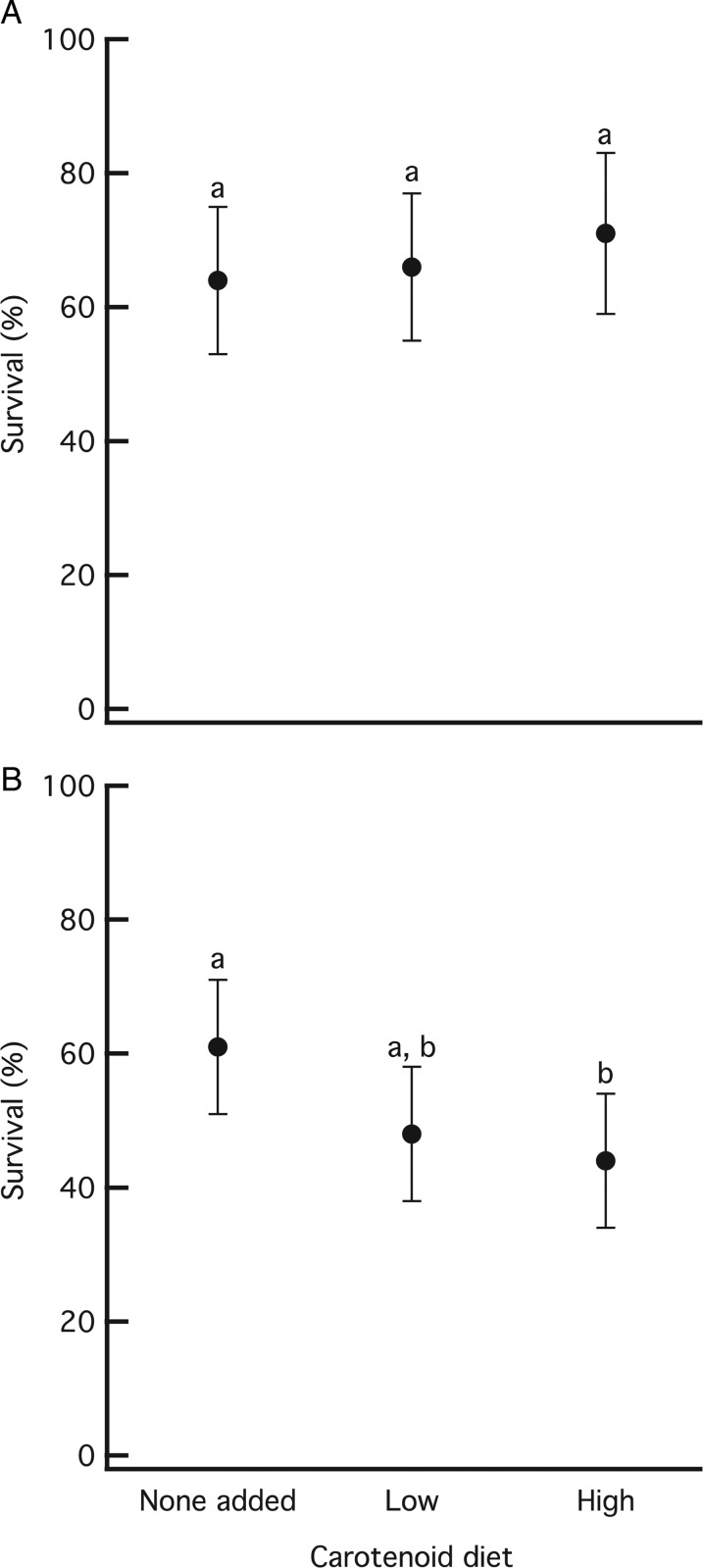
Survival of wood frogs (**A**) and gray treefrogs (**B**) after being raised to metamorphosis on three different carotenoid diets. Data are means ± 95% confidence intervals.

For survival to metamorphosis (Fig. 1), we found no effect of the carotenoid diets on wood frogs (Wald χ^2^ = 0.872, *P* = 0.647), but there was an effect on gray treefrogs (Wald χ^2^ = 6.508, *P* = 0.039). Gray treefrog survival decreased by nearly 20% when raised on a diet containing high carotenoids compared with a diet containing no added carotenoids (*P* = 0.038). This mortality was spread throughout the larval period. No other pairwise comparisons were significant (all *P* ≥ 0.114).

**Figure 2:**
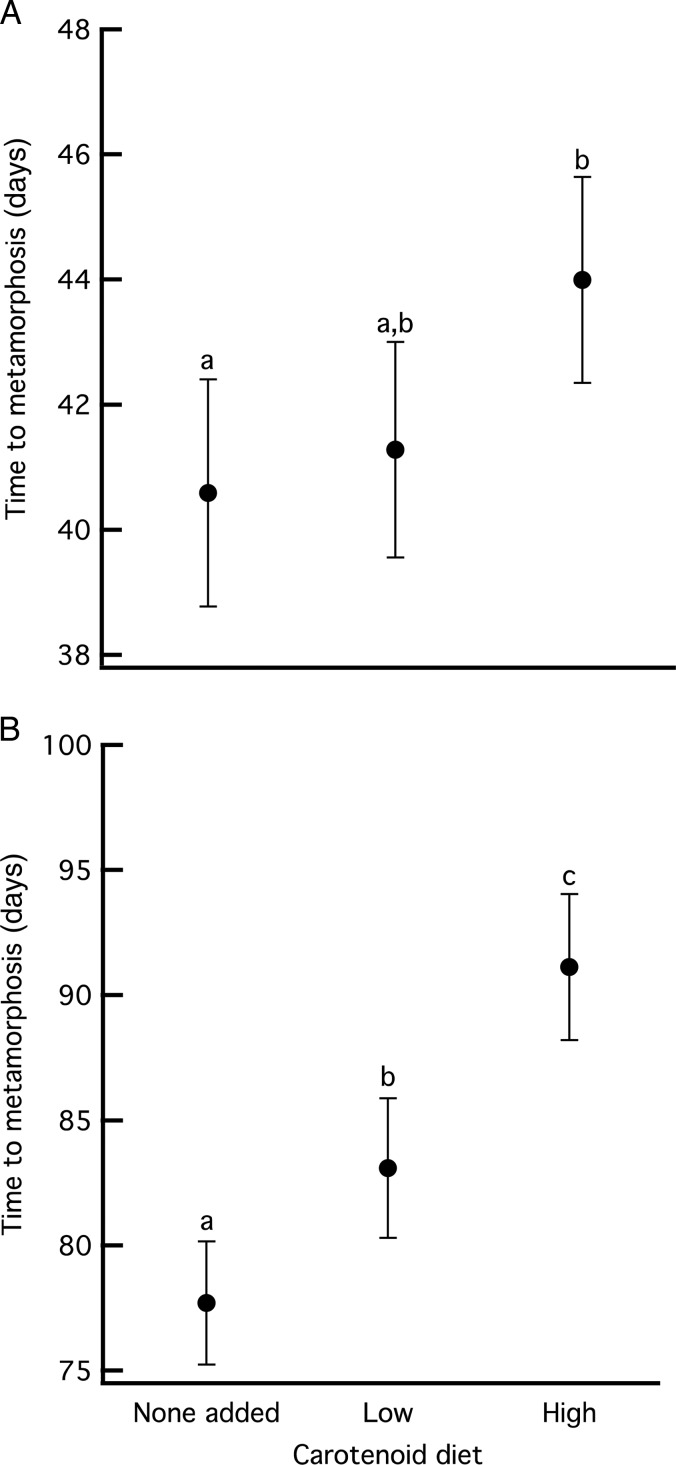
Time to metamorphosis of wood frogs (**A**) and gray treefrogs (**B**) after being raised to metamorphosis on three different carotenoid diets. Data are means ± 95% confidence intervals.

The carotenoid diets affected time to metamorphosis in both species (wood frogs, Wald χ^2^ = 8.498, *P* = 0.014; and gray treefrogs, Wald χ^2^ = 115.92, *P* < 0.001; Fig. 2). Wood frogs fed a diet containing high carotenoids took an average of 3 days longer to metamorphose than those fed on a diet containing low carotenoids or no added carotenoids (both *P* < 0.035). The latter two treatments did not differ (*P* = 0.493). Gray treefrogs fed a diet containing high carotenoids also had a longer time to metamorphosis. In comparison to diets with no added carotenoids, animals took an average of 5 days longer to metamorphose when reared on a low-carotenoid diet and 13 days longer when reared on a high-carotenoid diet (both *P* < 0.001). The latter two treatments also differed, with the high-carotenoid diet causing gray treefrogs to metamorphose 8 days later than the low-carotenoid diet (*P* < 0.001).

**Figure 3:**
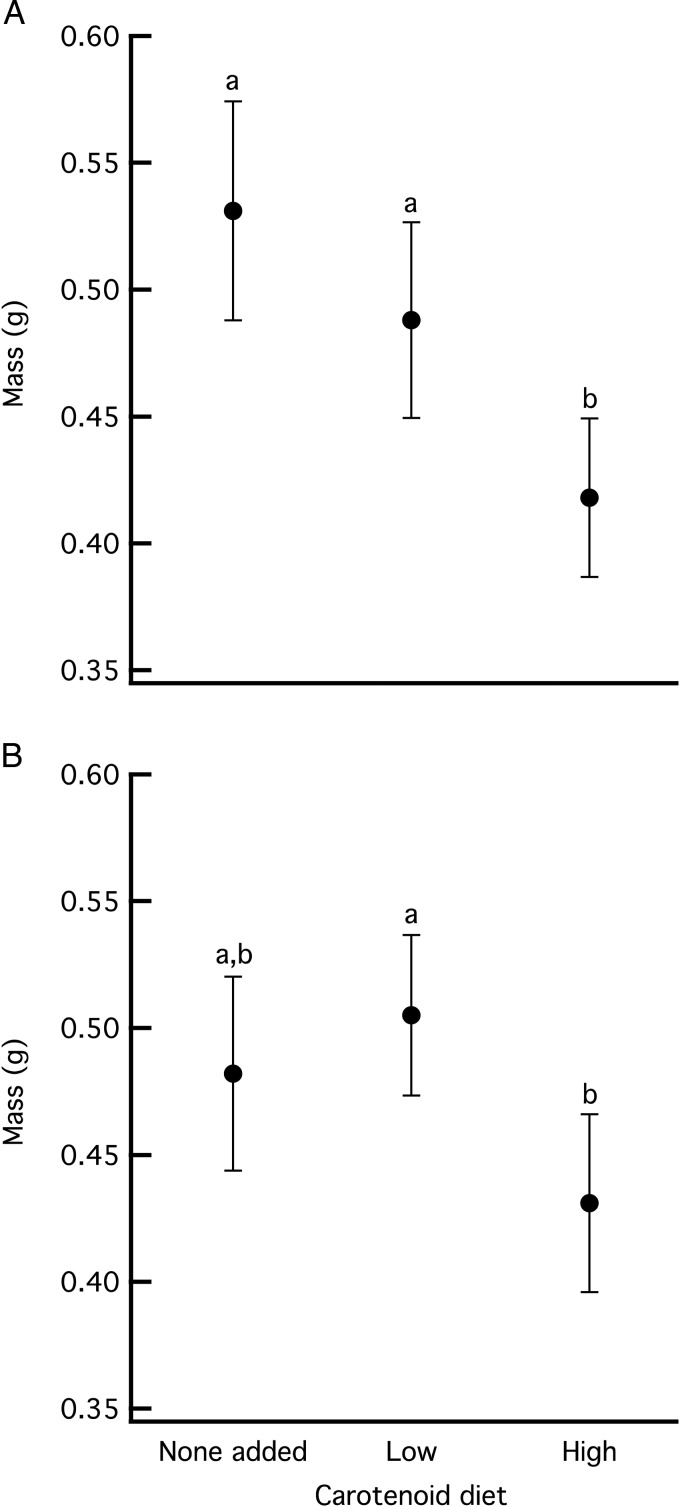
Mass of wood frog (**A**) and gray treefrog metamorphs (**B**) immediately before being exposed to the *Batrachochytrium dendrobatidis* (*Bd*) or no-*Bd* treatments. Data are means ± 95% confidence intervals.

The carotenoid diets also affected the mass of both species (wood frogs, *F*_2,107_ = 7.681, *P* = 0.001; and gray treefrogs, *F*_2,136_ = 4.052, *P* = 0.02; Fig. 3). Wood frog metamorphs reared on a high-carotenoid diet were 17% smaller than those reared on a low-carotenoid diet (*P* = 0.055) and 27% smaller than those reared on a diet with no added carotenoids (*P* < 0.001). The latter two treatments did not differ (*P* = 0.307). Gray treefrog metamorphs reared on a high-carotenoid diet were smaller than those reared on a low-carotenoid diet (*P* = 0.018) and tended to be smaller than those reared on a diet with no added carotenoids, although the difference was not significant (*P* = 0.135). The latter two treatments did not differ (*P* = 1.0).

### Effects of carotenoid diet and *B. dendrobatidis* on metamorph survival

After exposing the metamorphs to the presence or absence of *Bd* for 2 weeks, we examined metamorph survival (Table 1). For wood frogs, 59% survived in the absence of *Bd*, whereas 22% survived in the presence of *Bd*. The Cox regression model showed that the treatments affected survival in the experiment (omnibus test of model coefficients *P* < 0.001). Animals exposed to *Bd* were five times more likely to die than unexposed animals (i.e. the hazard ratio was 5.4), and larger individuals were less likely to die than smaller individuals. However, carotenoid diets had no effect on survival (i.e. there was no carotenoid diet or carotenoid diet-by-*Bd* exposure interaction).

**Table 1:** Results from the Cox regression model showing the effects of carotenoid diet, exposure to *Batrachochytrium dendrobatidis* and their interaction on the survival of wood frog metamorphs

	Wood frogs		
Source	χ^2^	d.f.	*P*-value
Carotenoid diet	0.97	2	0.616
*Bd* exposure	11.971	1	0.001
Interaction	0.637	2	0.727
Mass	6.415	1	0.011

Mass was included as a covariate in the model. The hazard ratio (95% confidence interval) for the effect of *Batrachochytrium dendrobatidis* (*Bd*) was 5.4 (2.1–14.0).

For gray treefrog survival, 94% survived in the absence of *Bd*, whereas 87% survived in the presence of *Bd.* The Cox regression model showed that the treatments had no effect on survival (omnibus test of model coefficients *P* = 0.22). Of the 139 metamorphs in the 14 day exposure, only 13 died (nine exposed to *Bd* and four not exposed to *Bd*).

### Effects of carotenoid diets on *B. dendrobatidis* infection loads

Our final analysis examined *Bd* infection loads in the metamorphs. In the ANOVA on wood frogs, we also found no effect on the carotenoid treatments on pathogen load (*F*_2,21_ = 0.584, *P* = 0.567); the mean number of genome equivalents ranged from 116 ± 35 to 186 ± 62. In the ANOVA on gray treefrogs, we found no effect of the carotenoid treatments on pathogen load (*F*_2,19_ = 0.774, *P* = 0.475); the mean number of genome equivalents (±1 SEM) ranged from 86 ± 27 to 158 ± 62. A subsequent power analysis showed that, given our sample size of eight swabbed animals and the variation in genome equivalents among tadpoles, mean differences between carotenoid treatments would have to be around 200–225 genome equivalents for wood frogs and gray treefrogs to achieve statistical significance (at a power of 0.8).

## Discussion

### Effects of carotenoids on larval amphibians

High-carotenoid diets had no effect on the survival to metamorphosis in wood frogs, but caused a 20% decline in gray treefrog survival. This was particularly surprising given the generally positive effects of carotenoids in a wide range of taxa (Olson and Owens, 1998; Pérez-Rodríguez, 2009; Simons *et al.*, 2012). Amphibian studies are rare, but two other species fed diets with supplemented carotenoids either exhibited no effect on survival or a small increase in survival (Ogilvy and Preziosi, 2012; Ogilvy *et al.*, 2012). Few studies of carotenoid diet manipulations track survival, but a study of three-spined sticklebacks (*Gasterosteus aculeatus*) observed increased survival in fish that were fed high-carotenoid diets (Pike *et al.*, 2007). These differences in survival responses may reflect the difference in carotenoid concentration, species differences in responses to carotenoids or differences in the ingredients used to manipulate carotenoid concentration.

The decline in the growth and development of the wood frogs and gray treefrogs was counter to our hypothesis that carotenoids would be beneficial. Among studies of amphibians that have manipulated carotenoid diets, Ogilvy *et al.* (2012) observed no effect on the growth of red-eyed treefrog tadpoles for several weeks post-metamorphosis. In a study using western clawed frogs, Ogilvy and Preziosi (2012) also found no effect of carotenoids on larval growth. In other vertebrate classes, there are few investigations of how carotenoid manipulations affect growth. In an experiment using great tit nestlings (*Parus major*) that were living in environments with high or low metal pollution, Eeva *et al.* (2009) found that adding carotenoids caused higher growth in the polluted environment but not in the unpolluted environment. One caveat to our work on growth and development is that animals were not weighed immediately after metamorphosis, but rather once they arrived at OSU before being used in the *Bd* challenge experiment. Thus, growth differences before and after metamorphosis may both have contributed to the overall mass differences on the day that the frogs were weighted.

We also found that both amphibian species exhibited slower development when fed diets high in carotenoids. Only two other studies have examined carotenoid effects on amphibian development. Ogilvy and Preziosi (2012) found that feeding western clawed frogs a diet high in carotenoids caused more rapid development than when fed a diet without added carotenoids. Dugas *et al.* (2013) found that female strawberry poison frogs, which produce trophic eggs to feed to their offspring, fed diets supplemented with carotenoids had higher reproductive success than those not fed supplemental carotenoids. This effect was driven by a combination of more successful embryonic development to the tadpole stage and more successful development of tadpoles to the metamorph stage. Negative effects of carotenoids on life-history traits are rare, and the few studies that we discovered are in birds (Costantini *et al*., 2007; Simons *et al*., 2014). Clearly, much more research needs to be conducted before we can develop generalities in growth and development responses to carotenoids.

Given that increased carotenoids caused one of our species to exhibit lower survival and both species to exhibit slower growth and development, one obvious question is whether the carotenoids we used are in some way toxic to gray treefrog tadpoles but not to wood frog tadpoles (note that neither species experienced mortality from carotenoids after metamorphosis). The formulation of carotenoids that we used was modelled to be similar to previous amphibian studies (Ogilvy and Preziosi, 2012; Ogilvy *et al.*, 2012). While those studies included β-carotene and our study included astaxanthin (which is a common component of aquatic animal tissues; Matsuno, 2001; Guerin *et al*., 2003; Gaillard *et al*., 2004), the effects of different types of carotenoids on the life history and immune function of animals are unknown. While it is known that astaxanthin is better at scavenging free radicals than β-carotene *in vitro* (Jørgensen and Skibsted, 1993), it is unknown whether astaxanthin is better at scavenging free radicals *in vivo*. Regardless, the carotenoid diets of Ogilvy and Preziosi (2012), which spanned the same range of concentrations (0–10 mg/g of total diet) as our study, did not have harmful effects on western clawed frogs. In studies using freshwater amphipods (*Hyalella* spp.), we have used carotenoid diets that were identical to those used for wood frogs and gray treefrogs and have found that the amphipods survived better on diets supplemented with carotenoids (R. D. Cothran, personal observation), so it seems unlikely that the carotenoid diets used in our experiment are generally toxic. They may simply be toxic to certain species of amphibians. This is an important discovery because, as noted by Ogilvy and Preziosi (2012), understanding the effect of carotenoids is a key issue when raising amphibians in captivity to save endangered species from extinction (Moore and Church, 2008).

### Effects of carotenoids on metamorphs exposed to *B. dendrobatidis*

When exposed to *Bd* after metamorphosis, we observed a dramatic difference in outcome; wood frogs died in large numbers when exposed to *Bd*, whereas gray treefrogs did not. Such species-level variation in susceptibility has been observed in previous experiments (Gervasi *et al*., 2013). Our results are consistent with a recent comparative study by Searle *et al.* (2011), who that found wood frogs exposed to *Bd* had a much higher hazard ratio (ratio ± SEM = 77.8 ± 0.7) than gray treefrogs (3.0 ± 0.3) because of species-level differences in tolerance. In addition, Searle *et al.* (2011) found that the risk of mortality declined with body mass of the metamorphs, a result that was also observed in wood frogs but not gray treefrogs in the present study.

A primary focus of our study was to investigate whether carotenoids in the diet throughout the tadpole and early metamorph stages could mitigate mortality caused by *Bd*. We were unable to assess this hypothesis in gray treefrogs, because they experienced such low mortality after exposure to *Bd*. However, the lack of a carotenoid effect on pathogen load suggests that carotenoids were not having any mitigating effect in this species. In contrast, the wood frogs experienced high mortality after exposure to *Bd*, and carotenoids had no effect on their pathogen load or their survival. This was a surprising result given the current paradigm that carotenoids are important in boosting the innate and acquired immune systems (Olson and Owens, 1998; Chew and Park, 2004; Duriancik *et al.*, 2010; Hall *et al.*, 2011). For example, diets higher in carotenoids can increase antioxidant defenses and lower parasitism in birds (Horak *et al*., 2001; Alonso-Alvarez *et al*., 2004; Hill and Farmer, 2005), lower rates of oxidative stress in fish (Pike *et al.*, 2007) and provide increased resistance to bacterial infections in amphipods (Babin *et al*., 2010). We found no evidence for a mitigating effect of carotenoids on *Bd* in amphibians, which may be because carotenoids do not boost the immune system of amphibians or they boost those components of the immune system that are not relevant to combating *Bd* infections. Alternatively, it may be that the immune system plays little or no role in combating *Bd* infections. However, the innate immune system appears to play a key role in producing skin peptides that possess antifungal properties (Rollins-Smith and Conlon, 2005; Woodhams *et al*., 2006), and the acquired immune system has been shown to play a role in responding to *Bd* infections in several species (reviewed by Richmond *et al*., 2009; Ramsey *et al*., 2010; Savage and Zamudio, 2011), although this is not a universal observation (Ribas *et al*., 2009; Cashins *et al*., 2013). Finally, there is the possibility that one might observe alternative outcomes at different concentrations of carotenoids than those used in the present study.

### Conclusions

Carotenoids have received a great deal of attention for improving immune systems and serving as indicators of overall animal condition. However, we know relatively little about the effect of carotenoids in amphibians beyond the fact that they can contribute to brighter colouration in some species. We discovered that high-carotenoid diets can decrease survival, development and growth and may not play a role in mitigating resistance or tolerance to the global pathogen, *Bd*. While our study is the first to examine this question in amphibians, should the result be generalizable to other amphibian species, it suggests that providing carotenoids to amphibians rescued from *Bd*-positive regions of the world and held in captivity will not necessarily provide increased protection from this deadly pathogen. Clearly, much more work needs to be conducted on other amphibian species to determine the generality of our findings.

## Supplementary material

Supplementary material is available at *Conservation Physiology* online.

## Funding

This research was funded by a National Science Foundation grant to R.A.R. and A.R.B. (DEB 07-16149, including an REU and RET supplement) and a National Graduate Women in Science Hartley Corporation Fellowship to S.S.G.

## Data accessibility

All data and metadata are available at http://dx.doi.org/10.5061/dryad.1qb31

## Supplementary Material

Supplementary Data
