# Supplementary material for: Carotenoids and amphibians: effects on life history and susceptibility to the infectious pathogen, Batrachochytrium dendrobatidis
Source: Conserv Physiol. 2015 Mar 13;3(1):cov005. doi: 10.1093/conphys/cov005 (PMC4778475; doi:10.1093/conphys/cov005)
Supplement: Supplementary Data [file cov005supp.doc]

**Digital appendix**

*Cricket maintenance*

Crickets fed a high carotenoid diet contain higher concentrations of carotenoids in their bodies (Ogilvy *et al.,* 2011; 2012). The crickets used to feed metamorphs were offspring from mothers that had been raised on the different carotenoid diets. Thus, the crickets were not simply gut-loaded with the different carotenoid diets, but were assimilating the different carotenoid diets throughout their life. Thus, tadpoles in our experiments were directly ingesting the different carotenoid diets and metamorphs were indirectly ingesting the same diets by eating the crickets that were reared on the carotenoid diets (Ogilvy *et al.,* 2011; 2012). Although we cannot directly compare the concentration of carotenoids in the tadpole food versus the metamorph food (i.e. the crickets), we can be certain that we maintained differences in carotenoid concentrations among the three carotenoid treatments to which the amphibians were exposed throughout their larval and early metamorphic lives.

Three separate terraria were used for the cricket carotenoid diet treatments. Each was 31 cm long x 20 cm tall x 15 cm deep with a volume of approximately 10 L. Each terrarium contained at most approximately 1,500 1-wk-old crickets. Each week approximately 1,000 additional crickets were added to the terraria (obtained from sunshinemealworms.com). Crickets were provided with approximately 1 g of crushed carotenoid wafers daily (see earlier description), which proved to be an *ad libitum* diet. The cricket food was also present in the metamorph tubs to ensure that any crickets not immediately consumed by the metamorphs would retain their carotenoid levels. Crickets were not provided any other food source. Water was checked in the terraria daily and changed if necessary.

*Bd preparation and inoculation*

*Batrachochytrium dendrobatidis* isolate, JEL 274 (originally isolated from *A. boreas* in Colorado; Annis et al. 2004), was cultured on 100 mm X 15 mm tryptone agar plates and allowed to grow for 6 d at 20 to 22°C before inoculation of animals. Zoospores were harvested by flooding agar plates with 15 ml of dechlorinated water and scraping the surface of the agar before pooling the inoculums of several (5 to 10) plates.

Experimental animals were exposed to 10 ml of pooled inoculation broth at a density of 10,000 zoospores/ml, which resulted in a total exposure to 100,000 zoospores. Previous studies have found that this level of exposure causes Bd loads similar to the current study and causes amphibian mortality (Gervasi *et al.,* 2013). All zoospore counts were determined by hemocytometer from pooled inoculation broth. Zoospore broth was transferred to individual petri dishes, which already contained 15 ml of dechlorinated water. Control animals were exposed to the same volume of sham inoculation (created from pathogen-free tryptone-agar plates).

**Appendix literature cited**

Annis SL, Dastoor FP, Ziel H, Daszak P, Longcore JE (2004) A DNA-based assay identifies *Batrachochytrium dendrobatidis* in amphibians. *J Wildl Dis* 40:420-428.

Babin A, Biard C, Moret, Y (2010) Dietary supplementation with carotenoids improves immunity without increasing its costs in a crustacean. *Am Nat* 176:234-241.

Gervasi SS, Urbina J, Hua J, Chestnut T, Relyea RA, Blaustein AR (2013) Experimental evidence for differential susceptibility to the amphibian chytrid fungus (Batrachochytrium dendrobatidis) in the American bullfrog (Lithobates catesbeianus). *EcoHealth* 10:166-171.

Ogilvy VA, Fidgett L, Preziosi RF (2011) Differences in carotenoid accumulation among three feeder-cricket species: Implications for carotenoid delivery to captive insectivores. *Zoo Biol* 30:1-9.

Ogilvy V, Preziosi RF, Fidgett AL (2012) A brighter future for frogs? The influence of carotenoids on the health, development and reproductive success of the red-eyed tree frog. *Anim Conserv* 16:480-488.

Table A1. Species information for the wood frog and gray treefrog experiments. Information includes where the animals were collected, how many egg masses were collected, and the initial mass and Gosner stage (Gosner 1960) of the tadpoles at the start of the experiment.

| **Species** | **Pond**  **latitude** | **Pond**  **longitude** | **Number**  **of egg**  **masses** | **Initial mean**  **mass ± 1 SD;**  **(mg)** | **Initial**  **Gosner**  **stage** |
| --- | --- | --- | --- | --- | --- |
| Wood frogs | 41.8414 | -80.2400 | 7 | 66 ± 14 | 27-28 |
| Gray treefrogs | 41.5694 | -80.4564 | 15 | 10 ± 4 | 25 |

Table A2. Ingredients and quantities used to make the three carotenoid diets. The CAROPHYLL Pink® contained 10% astaxanthin whereas the FloraGLO contained 5% lutein, so the carotenoids were present in a 4:1 ratio of astaxanthin:lutein, following the diets used by Babin et al. (2010).

|  | **Carotenoid treatment**  **(mg of total carotenoids per g of diet)** | | |
| --- | --- | --- | --- |
| **Ingredients** | None (0) | Low (1) | High (10) |
| *Water (g)* | 78 | 78 | 78 |
| *Fishmeal (g)* | 40 | 40 | 40 |
| *Wheat flour (g)* | 30 | 30 | 30 |
| *Rice flour (g)* | 30 | 30 | 30 |
| *Vitamins (g)* | 2 | 2 | 2 |
| *CAROPHYLL® Pink (g)* | 0 | 1.44 | 14.4 |
| *FloraGLO® (g)* | 0 | 0.72 | 7.2 |

CAROPHYLL® Pink contains astaxanthin and the following “inert” ingredients: lignosulfonate, corn oil and ethoxyquin.

FloraGLO® contains lutein and the following “inert” ingredients: crystalline lutein, sucrose and tapioca starch.

Table A3. The composition of the vitamins used in the amphibian diets.

|  |  |
| --- | --- |
| **Ingredients** | **Amount (/227)** |
| Vitamin A | 1432 USP |
| Vitamin E | 143.2 IU |
| Vitamin B12 | 0.044 mmol |
| Vitamin D3 | 286 USP |
| Vitamin K | 3.50 mg |
| p-Panthothenic Acid | 72.7 mg |
| Choline | 11.5 mg |
| Pyridoxine | 11.5 mg |
| Ascorbic acid | 384 mg |
| BHT | 0.88 mg |
| Riboflavin | 14.3 mg |
| Niacin | 85.9 mg |
| Thiamine | 11.4 mg |
| Folic Acid | 3.43 ng |
| Biotin | 0.17 mg |
| Inositol | 57.3 mg |
